# Trends in Proportion of Medicare Part D Claims Subject to 340B Discounts, 2013-2020

**DOI:** 10.1001/jamahealthforum.2023.4091

**Published:** 2023-11-17

**Authors:** Sean Dickson, Nico Gabriel, Inmaculada Hernandez

**Affiliations:** 1 West Health Policy Center, Washington, DC; 2 Division of Clinical Pharmacy, Skaggs School of Pharmacy and Pharmaceutical Sciences, University of California, San Diego, La Jolla

## Abstract

**Importance:**

Despite controversy surrounding the 340B program, no study has analyzed trends in the proportion of Medicare Part D pharmacy claims eligible for 340B discounts.

**Objective:**

To describe trends in the proportion of Medicare Part D claims that are prescribed by 340B-affiliated clinicians and filled in 340B pharmacies.

**Design and Setting:**

This longitudinal, retrospective cohort study included 2013 to 2020 claims data from a 5% random sample of Medicare Part D beneficiaries from the Centers for Medicare & Medicaid Services and 6292 nine-digit national drug codes that were used by at least 1000 Part D beneficiaries in a given year. Data analysis was completed from November 2022 to April 2023.

**Main Outcomes and Measures:**

For each drug and year, there were 3 outcomes: (1) proportion of total Part D claims that were prescribed by a 340B-affiliated clinician; (2) proportion of claims prescribed by a 340B-affiliated clinician that were filled in a 340B pharmacy; and (3) proportion of total Part D claims under the 340B program (ie, prescribed by a 340B-affiliated clinician and filled in a 340B pharmacy).

**Results:**

The proportion of prescriptions written by a 340B-affiliated clinician doubled from 9.4% in 2013 to 19.3% in 2020. The capture of 340B prescriptions by 340B pharmacies, defined as the proportion of claims prescribed by 340B-affiliated clinicians that were filled by 340B pharmacies, increased from 18.4% in 2013 to 49.9% in 2020. As a result, the total proportion of 340B claims in Part D increased from 1.7% in 2013 to 9.6% in 2020. Rates of 340B prescribing and capture increased consistently across therapeutic classes. In 2020, the antiviral therapeutic class was the class with the largest proportion of 340B claims (16.1%), followed by targeted antineoplastics (15.7%).

**Conclusions and Relevance:**

This cohort study demonstrated that from 2013 to 2020, the share of Medicare Part D claims prescribed by a 340B-affiliated clinician increased; however, the rate at which 340B-eligible prescriptions were filled at 340B pharmacies increased at a faster rate, driving the overall increase in 340B claims. Despite these trends, only half of 340B-eligible prescriptions were subject to the 340B discount in 2020.

## Key Points

**Question:**

How has the proportion of Medicare Part D claims eligible for 340B discounts changed over time?

**Findings:**

In this cohort study of 2013 to 2020 Medicare Part D claims data, the proportion of 340B claims increased from 1.7% in 2013 to 9.6% in 2020. This was driven by increases in prescribing by 340B-affiliated clinicians (9.4% in 2013 vs 19.3% in 2020) and capture of prescriptions by 340B pharmacies (18.4% in 2013 vs 49.9% in 2020).

**Meaning:**

Despite the expansion of the 340B program, less than half of the claims prescribed by 340B-affiliated clinicians are captured by 340B pharmacies.

## Teaser

This cohort study describes trends in the proportion of Medicare Part D claims that are prescribed by 340B-affiliated clinicians and filled in 340B pharmacies.

## Introduction

The 340B Drug Discount Program (hereafter, 340B program) allows certain federally designated hospitals and clinics to acquire outpatient prescription drugs at a discounted price while receiving customary reimbursement from insurers at an undiscounted price, generating additional revenue.^1^ The 340B program was established in 1992 following the creation of the Medicaid Drug Rebate Program,^2^ which required pharmaceutical manufacturers to provide rebates to state Medicaid programs to offset prescription drug spending. These Medicaid rebates are calculated as the sum of a base rebate and an inflation penalty. For branded drugs, the base rebate is calculated as the greater of 23.1% of the average manufacturer price or the greatest discount that the drug manufacturer offers to commercial customers (called the “best price” requirement). The inflation rebate is a penalty that offsets price increases above the rate of inflation.^3^ Following the establishment of the best price requirement, manufacturers reduced or eliminated voluntary discounts to safety net and charitable health care providers to avoid increasing their Medicaid rebate liability.^4,5^ In response, Congress established the 340B program, which required drug manufacturers to extend the Medicaid discount to certain federally designated health care providers in exchange for exempting these and other discounts from the Medicaid best price requirement. Notably, the 340B discount also includes the Medicaid program’s inflation penalty discount.^6^

The plurality of 340B covered entities (46.4% in 2023^7^) is disproportionate share hospitals. Disproportionate share hospitals qualify for 340B discounts based on the share of their patient population eligible for Medicaid, nonprofit status, and contracting with local governments to provide services.^8^ Other 340B entities^9^ include children’s hospitals, free-standing cancer hospitals, critical access hospitals, federally qualified health centers, Ryan White HIV/AIDS clinics, family planning clinics, and sexually transmitted disease clinics, which are eligible based on their receipt of designated federal funding. Beyond these eligibility criteria, additional requirements designate which patients are eligible to be dispensed drugs acquired at a discount.^10^

To be subject to 340B discounts, prescriptions need to be written by a 340B-affiliated clinician and dispensed from either an on-premise pharmacy or a contract pharmacy on behalf of the 340B covered entity. These arrangements with contract pharmacies allow patients to fill their prescription at a preferred pharmacy (often a chain pharmacy) while allowing the 340B entity to benefit from the 340B discount.^11,12^ Absent this arrangement, only prescriptions dispensed from an on-premise pharmacy of the 340B entity would be eligible for 340B discounts.

340B-eligible entities are not required to pass along discounts obtained under the program to insurers or patients; instead, the program was intended to generate revenue for these entities to “stretch scarce federal resources as far as possible, reaching more eligible patients and providing more comprehensive services.”^13^ The use of these revenues has generated controversy—while 340B entities eligible through receipt of federal grants are required to use 340B discounts for the same purposes as their federal grants, stretching federal dollars, hospitals do not have the same limits on how they may use 340B revenues.^14^ Drug manufacturers have argued that hospitals should be required to pass discounts to patients^15,16^ or provide additional charity care^17^ with 340B revenues.

Given the intense controversy over the 340B program, few studies have analyzed trends in 340B program eligibility and discount magnitude. Previous work has considered how many prescriptions were written by 340B-eligible prescribers but did not assess whether the prescription was filled at an on-site or contract pharmacy, which is required for a prescription to generate the 340B discount.^18^ Other relevant work has measured growth in 340B eligibility,^19,20,21^ considered the association between 340B eligibility and provision of charity care,^22,23^ or compared prescribing patterns between 340B-affiliated and nonaffiliated clinicians.^24,25,26^ To our knowledge, no study has measured trends in the share of pharmacy claims that are prescribed by 340B-affiliated clinicians and filled at 340B pharmacies and, therefore, subject to a 340B discount. This information is relevant to inform policymakers as they assess competing claims from drug manufacturers and 340B entities while considering policy reforms.

## Methods

### Data Sources and Study Sample

We obtained 2013 to 2020 claims data from a 5% random sample of Medicare Part D beneficiaries from the Centers for Medicare & Medicaid Services. Every year, we selected 9-digit national drug codes (hereafter, drugs) that were used by at least 1000 Part D beneficiaries in the 5% random sample (n = 6292 drugs). Data analysis was completed from November 2022 to April 2023. The institutional review board at the University of California, San Diego, declared the study exempt from review because deidentified data were used in analyses. We followed the Strengthening the Reporting of Observational Studies in Epidemiology (STROBE) reporting guidelines in the preparation of the article.

### Outcome Variables

We specified 3 primary outcomes. All outcomes were estimated for each drug and year. The first outcome represented 340B prescribing, defined as the number of claims prescribed by a 340B-affiliated clinician divided by the total number of Part D claims for a given drug. The second outcome represented the capture of 340B prescriptions by 340B pharmacies, defined as the number of claims filled by a 340B pharmacy among the claims prescribed by a 340B-affiliated clinician. The third outcome represented whether a prescription was subject to a 340B discount (ie, whether a prescription was prescribed by a 340B-affiliated clinician and filled by a 340B pharmacy). The third outcome was defined as the number of claims prescribed by a 340B-affiliated clinician and filled by a 340B pharmacy divided by the number of Part D claims for a given drug.

We used previously published methods to identify claims prescribed by 340B-affiliated clinicians and filled by 340B pharmacies.^19,27,28,29,30,31,32,33,34^ In brief, to identify claims prescribed by a 340B-affiliated clinician (outcome 1), we address matched the 340B covered entity file for each year to the Medicare Part D prescriber utilization file.^13,35^ This enabled us to derive a list of National Provider Identifiers (NPIs) associated with 340B covered entities, including those practicing at off-site locations affiliated with a covered entity. We linked this list to the Part D claims using the NPI to identify claims prescribed by 340B-affiliated clinicians. We followed a similar process to identify the capture of 340B prescriptions by 340B pharmacies (outcome 2). First, we address matched the 340B contract pharmacy file to dataQ (National Council for Prescription Drug Programs), a data resource that contains information for all operating pharmacies in the US, including address and NPI.^36^ This matching enabled us to derive a list of pharmacy NPIs that represented 340B contract pharmacies. We linked this list to the Part D claims using the NPI of the dispensing pharmacy and identified claims dispensed by 340B contract pharmacies. We required the claim to be filled within the dates that the 340B contract was active to be considered reimbursed by a 340B pharmacy. Finally, outcome 3 was defined as those prescriptions that met both outcome 1 (prescribed by a 340B-affiliated clinician) and outcome 2 (filled in a 340B pharmacy).

### Statistical Analysis

We reported the outcomes by year for the overall sample and for subgroups defined by the Uniform System of Classification therapeutic class.^37^ We also reported outcomes for the top 10 drugs by Medicare Part D spending in 2020, identified using the Centers for Medicare & Medicaid Services Medicare spending dashboard.^38^ Statistical analyses were performed in SAS, version 9.4 (SAS Institute Inc).

## Results

### Overall Sample

**Figure 1.**
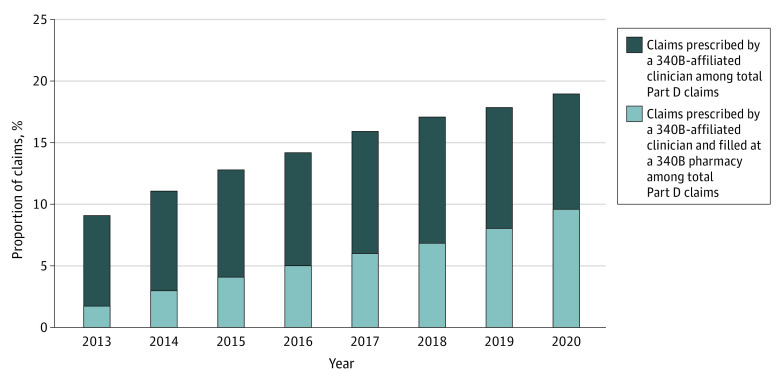
Trend in Share of Medicare Part D Prescriptions Written by a 340B-Affiliated Clinician and Filled at a 340B Pharmacy, 2013-2020 Calculated from a 5% sample of Medicare Part D claims data and a 340B covered entity database. Over the period, the share of Part D claims written by a 340B-affiliated clinician grew more slowly than the rate at which 340B-eligible prescriptions were filled in 340B pharmacies.

The proportion of prescriptions written by a 340B-affiliated clinician doubled from 9.4% in 2013 to 19.3% in 2020 (Figure 1). The capture of 340B prescriptions by 340B pharmacies, defined as the proportion of claims prescribed by 340B-affiliated clinicians that were filled by 340B pharmacies, increased from 18.4% in 2013 to 49.9% in 2020 (Table). As a result, the proportion of Part D claims subject to a 340B discount (prescribed by a 340B-affiliated clinician and filled at a 340B pharmacy) increased from 1.7% in 2013 to 9.6% in 2020 (Figure 1).

**Table.** Medicare Part D 340B Claim Eligibility of Therapeutic Class by Prescriber, Pharmacy, and Overall, 2013 and 2020

Therapeutic class	Claims, %^a^					
	Prescribed by 340B-affiliated clinician		Prescribed by 340B-affiliated clinician that were filled at 340B pharmacy		Prescribed by 340B affiliated clinician and filled at 340B pharmacy	
	2013	2020	2013	2020	2013	2020
Overall sample	9.4	19.3	18.4	49.9	1.7	9.6
Classes with >100 000 claims in 2020						
Antivirals	21.0	28.1	27.1	57.3	5.7	16.1
Antineoplastic targeted therapy	13.2	31.3	17.0	50.0	2.2	15.7
Antinauseants	11.3	22.9	19.5	52.2	2.2	12.0
Antineoplastic chemotherapy	9.4	21.6	18.3	52.4	1.7	11.3
Respiratory therapy	10.5	21.3	19.2	52.2	2.0	11.1
Cardiac agents	9.6	22.7	16.9	48.6	1.6	11.0
Antimalarials	10.2	20.5	18.6	51.4	1.9	10.5
Diuretics and aquaretics	10.4	21.1	17.8	49.4	1.9	10.4
Hemostatic modifiers	9.2	21.8	17.8	47.5	1.6	10.3
Hormones	9.6	19.6	19.5	52.6	1.9	10.3
Diabetes therapy	10.2	19.9	18.9	51.0	1.9	10.2
Musculoskeletal	9.1	19.4	18.8	52.0	1.7	10.1
Analgesics	9.9	18.7	20.5	53.8	2.0	10.1
Vascular agents	9.6	19.8	18.1	50.3	1.7	10.0
Antiarthritics	9.3	18.7	19.4	51.9	1.8	9.7
Nutrients and supplements	9.3	20.5	17.4	46.8	1.6	9.6
Antihyperlipidemic agents	9.2	19.0	18.1	50.2	1.7	9.5
Gastrointestinal agents	9.6	19.2	18.0	49.3	1.7	9.5
Anti-infectives, systemic	9.1	17.8	20.3	53.1	1.9	9.4
Genitourinary agents	9.0	19.2	17.6	48.3	1.6	9.3
Laxatives	9.8	19.5	16.7	47.6	1.6	9.3%
Antifungal agents	8.7	17.7	19.4	50.8	1.7	9.
Neurological/neuromuscular disorder agents	9.1	19.3	17.1	46.2	1.6	8.9
Thyroid therapy	8.5	18.0	17.3	48.9	1.5	8.8
Psychotherapeutic drugs	8.9	18.1	17.9	48.0	1.6	8.7
Sedatives and hypnotics	7.7	15.0	20.4	54.3	1.6	8.2
Allergy/cold preparations	7.4	16.2	17.3	48.8	1.3	7.9
Dermatologicals	7.7	15.6	17.9	47.5	1.4	7.4
Ophthalmic preparations	4.9	11.6	15.5	46.2	0.8	5.4
Antiseptics	8.0	15.0	15.5	34.2	1.2	5.1

^a^ Calculated from a 5% sample of Medicare Part D claims data and a 340B covered entity database. Claims prescribed by a 340B-affiliated clinician are a percentage of all claims in the therapeutic class, while 340B-eligible claims filled at a 340B pharmacy are a percentage of the claims prescribed by a 340B-affiliated clinician. Overall 340B claims are the product of the prior 2 columns, representing the percentage of total claims in the therapeutic class prescribed by a 340B-affiliated clinician and filled at a 340B pharmacy.

### Results by Therapeutic Class

**Figure 2.**
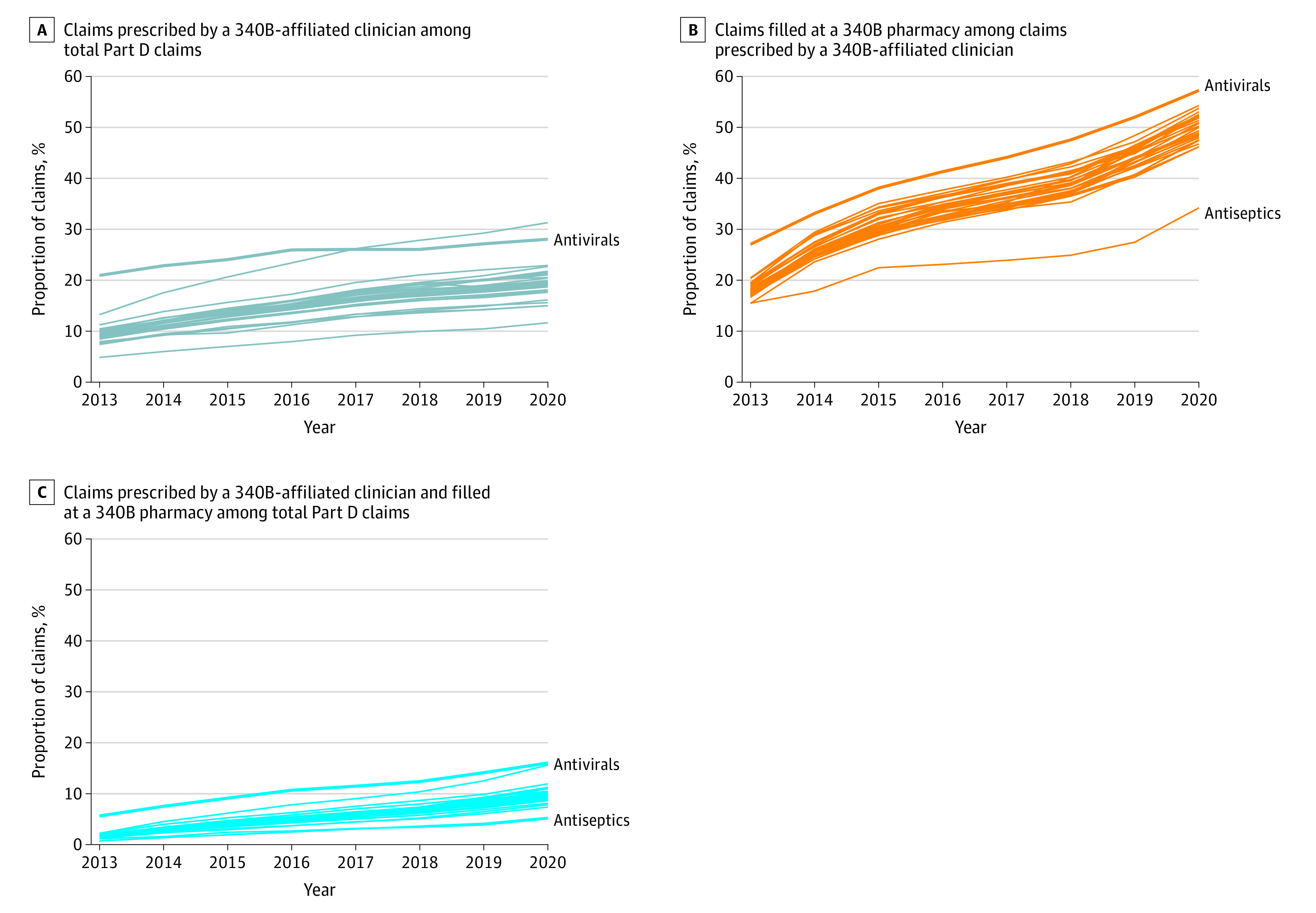
Trends in 340B Claim Eligibility, Fill Rates at 340B Pharmacies, and Overall 340B Share in Medicare Part D for High-Use Therapeutic Classes, 2013-2020 Calculated from a 5% sample of Medicare Part D claims data and a 340B covered entity database. Included therapeutic classes are those with greater than 100 000 Medicare Part D claims in 2020. A full list of classes can be found in the Table. Notably outlying classes are labeled.

Figure 2 and the Table show trends in 340B prescribing and filling trends for the 30 therapeutic classes with at least 100 000 Part D claims in the 5% random sample in 2020. Results for the remaining therapeutic classes are summarized in eTable 1 in Supplement 1. Rates of 340B prescribing and capture increased in parallel across therapeutic classes, following the trend observed for the overall sample. Consistently across the study period, the antiviral class was the therapeutic class with the largest proportion of claims prescribed by 340B-affiliated clinicians (28.1% in 2020), subsequently filled in 340B pharmacies (57.3% of prescriptions from 340B-affiliated clinicians in 2020), and subject to a 340B discount (16.1% of claims in the class in 2020), followed by targeted antineoplastics. Antiseptics and ophthalmic preparations were the classes with the lowest proportion of claims subject to a 340B discount (5.1% and 5.4% in 2020, respectively).

### Results for Top-Spending Drugs

**Figure 3.**
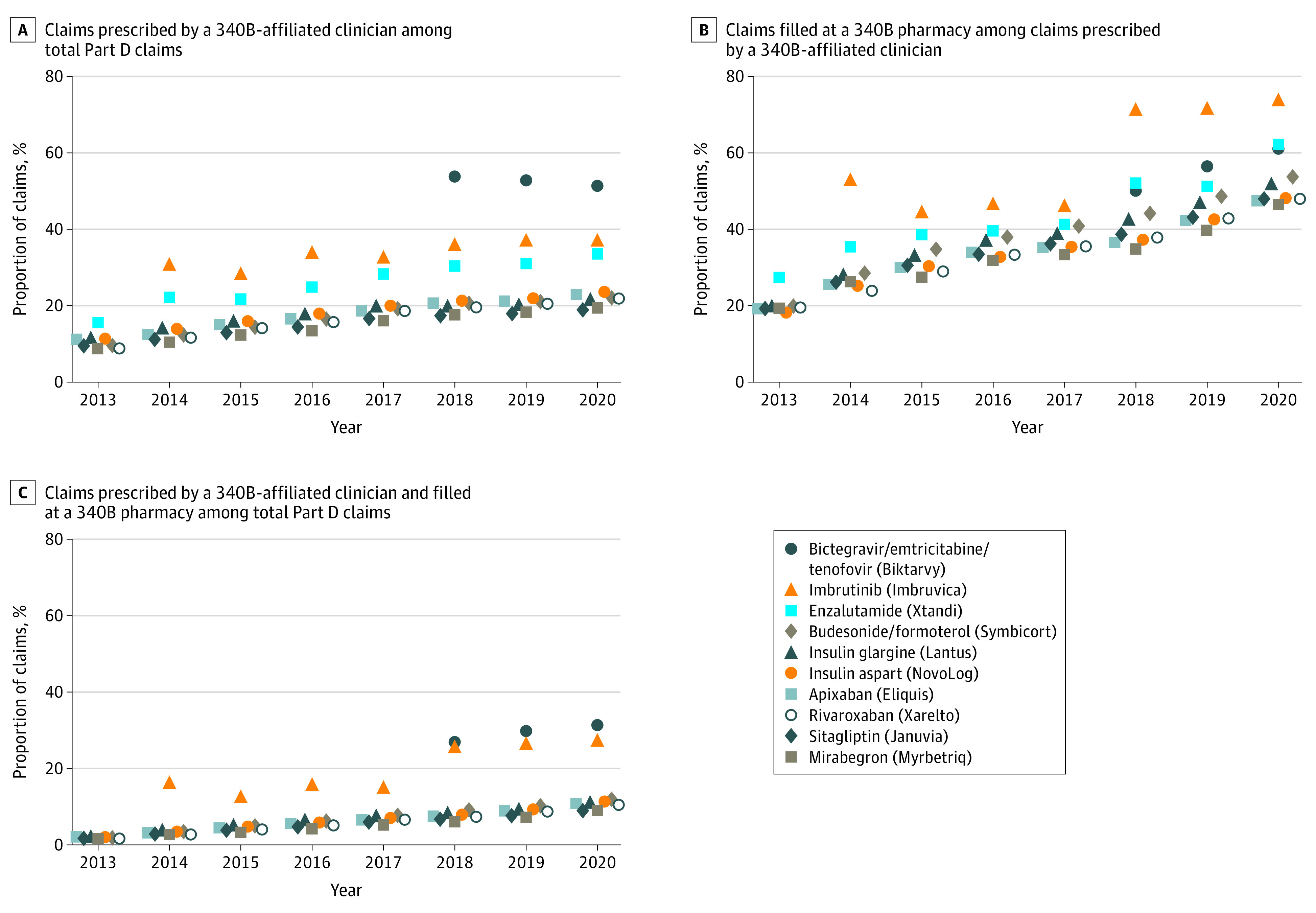
Trends in 340B Claim Eligibility, Fill Rates at 340B Pharmacies, and Overall 340B Share in Medicare Part D for High-Spend Drugs, 2013-2020 Calculated from a 5% sample of Medicare Part D claims data and a 340B covered entity database. Included drugs are the top 10 drugs by undiscounted Medicare Part D spending in 2020, as reported in the Medicare Part D dashboard. The 2018 discontinuity for Imbruvica (AbbVie) in the rate at which 340B-eligible claims were filled at 340B pharmacies may be attributable to changes in the formulation and limited distribution mechanism introduced that year.

Figure 3 and eTable 2 in Supplement 1 show trends in 340B prescribing and filling for the top 10 drugs by Medicare Part D spending in 2020. The HIV treatment bictegravir/emtricitabine/tenofovir alafenamide (Biktarvy) was the only top-spend drug with more than 50% of claims prescribed by 340B-affiliated clinicians (Figure 3A). The next top-spend drugs with the largest proportion of claims prescribed by 340B-affiliated clinicians were the cancer therapies imbrutinib (Imbruvica) and enzalutamide (Xtandi). For the remaining top-spending drugs, rates of 340B prescribing increased following the trend observed for the overall sample, from around 9% to 11% in 2013 to 18% to 23% in 2020.

With the exception of imbrutinib, which was the top-spend drug with the largest capture of claims by 340B pharmacies (73.9% in 2020), rates of prescription filling by 340B pharmacies increased following the results observed for the overall sample, from 18% to 20% in 2013 to 46% to 62% in 2020 (Figure 3B). As a consequence of these trends in 340B prescribing and capture, the HIV treatment bictegravir/emtricitabine/tenofovir alafenamide was the top-spend drug with the highest proportion of 340B claims subject to a discount (31.4% in 2020), followed by imbrutinib and enzalutamide (Figure 3C). With the exception of these 3 drugs, rates of claims subject to 340B discounts increased following the trends observed for the overall sample, from 1% to 2% in 2013 to 9% to 12% in 2020.

## Discussion

From 2013 to 2020, the share of Medicare Part D prescriptions that were subject to a 340B discount increased from 1.7% to 9.6%. While this growth was driven in part by the doubling of prescriptions written by 340B-affiliated clinicians (from 9.4% to 19.3%), the increase was primarily driven by the greater capture of 340B-eligible prescriptions by 340B pharmacies, which increased from 18.4% in 2013 to 49.9% in 2020. Despite this large increase, 340B covered entities are still only recouping half of the 340B revenue for which they are eligible.

To our knowledge, this study is the first to document trends in the share of prescriptions subject to 340B discounts. Although the results based on Medicare Part D data may not generalize to the overall population, the evidence is a major contribution to the ongoing controversy on the growth of the 340B program. The present data demonstrate empirically for the first time, to our knowledge, that the overall increase in prescriptions subject to 340B discounts is associated with the increasing capture of prescriptions by 340B pharmacies—from 2009 to 2022, the number of contract pharmacy arrangements increased from fewer than 1000 to 25 775.^39^ The growth in prescriptions originating from 340B covered entities may be due to increased hospital consolidation over the period, as the share of physicians employed by hospitals or corporate entities increased from 26% to 69% from 2012 to 2020.^40,41^ By expanding outpatient departments to locations that were traditionally physician offices, 340B hospitals increased the share of prescriptions that are 340B eligible.

The growth of contract pharmacies was the subject of intense scrutiny by manufacturers,^42^ which ultimately culminated with manufacturers’ decisions to limit the provision of 340B-discounted products to contract pharmacies.^43^ Because these manufacturer limits postdated the present study period, we were not able to estimate how they may have affected the proportion of Part D claims eligible for 340B discounts. Future research using more recent data should estimate the effect of manufacturer limits to contract pharmacies while accounting for the variability of restrictions over time and across manufacturers in terms of number of contract pharmacies authorized, types of covered entities the restrictions applied to, and exception criteria. For example, Eli Lilly first limited the distribution of 340B-discounted products with the exception of insulin to a single contract pharmacy for covered entities without an in-house pharmacy,^44^ then revised the policy to allow an unlimited number of contract pharmacies as long as the covered entity shared claims data for the contract pharmacy orders.^16^ In 2021, Novo Nordisk limited the provision of 340B products to 1 contract pharmacy for 340B hospitals without an in-house pharmacy^45^; in February 2022, it revised the policy to allow 2 contract pharmacies—a specialty and a retail pharmacy.^46^ In January 2023, Novo Nordisk announced that it would enable an unlimited number of contract pharmacies as long as the covered entity would share claims data for the contract pharmacy orders,^47^ then clarified in July 2023 that the waiver of the restriction through the sharing of claims only applied to contract pharmacies owned by the covered entity.^48^

Manufacturers’ issuance of restrictions in the provision of 340B-discounted products to contract pharmacies was found by the Health Resources and Services Administration in violation of the 340B statue and challenged in court. Several cases are pending resolution in appellate courts. If appellate courts, or ultimately the Supreme Court, were to side with manufacturers, as the US Court of Appeals for the Third Circuit did in early 2023,^49^ the 340B program would see a major reversal in the trends observed in this study period. If, however, the agency’s interpretation of the 340B statute was upheld, the proportion of claims eligible to 340B discounts would soon approximate that estimated in the latter years of the present study period.

At the therapeutic class level, the distribution of 340B eligibility is consistent with prior research,^30^ where antiviral therapies and cancer therapies have the greatest 340B eligibility and ophthalmic agents have the least. This distribution likely stems from the practice location associated with different morbidities—HIV and cancer are more likely to be treated in specialty clinics or hospital outpatient departments that are 340B eligible, while ocular conditions may be treated by general practitioners or ophthalmologists in private, nonhospital-affiliated offices. This distribution is also seen at the drug level among the highest-spend Part D drugs, where the antiretroviral bictegravir/emtricitabine/tenofovir alafenamide (Biktarvy [Gilead]) and the cancer therapies imbrutinib (Imbruvica [AbbVie]) and enzalutamide (Xtandi [Astellas]) have the greatest 340B prescribing and fill rates. Notably, following changes^50^ to the limited distribution mechanism in 2018, the share of 340B-eligible imbrutinib prescriptions that were filled at a 340B pharmacy increased, suggesting that the change in the distribution chain may have affected how and where prescriptions were dispensed.

The rising share of claims subject to 340B discounts generates indirect benefits beyond the provision of care to the underserved population subsidized by 340B discounts. One such indirect benefit is the likely reduction in drug price growth, as previous work has demonstrated that 340B eligibility is inversely associated with increases in drug prices.^19^ Specifically, a 10% difference in the share of 340B-eligible claims from one drug to another is associated with a 1.1%-lower price increase. Given the substantial increase in 340B prescriptions filled observed in the current study, the association of increasing 340B discounts with tempering drug price increases may be even greater. On the other hand, 340B discounts have been cited as a contributor toward drug shortages of inexpensive injectable drugs with a high 340B share, such as generic cancer therapies.^51^ 340B discounts have also been suggested as a driver of hospital and health care provider consolidation, which can increase costs for medical services.^20^

In assessing the 340B program, it is important to recognize that the generation of 340B discounts does not increase national health expenditures. Absent the 340B program, the covered entity would acquire the drug at the undiscounted price, and the reimbursement from the insurer would be the same. Discounts under the 340B program, then, should be understood as a subsidy from drug manufacturers to certain entities.

### Limitations

This analysis is subject to several limitations. First, the use of the 5% Medicare Part D sample and the nature of matching NPIs across multiple data sets may result in errors or distributions that do not reflect the entire Medicare Part D population; however, this would be unlikely to affect the temporal trends and relative differences across therapeutic classes and drugs. Second, the results may not generalize to the overall population because of the use of Medicare claims data and differences in health care provider and drug mix across populations. Third, as previously stated, the findings from 2013 to 2020 may not generalize to the present time due to manufacturer restrictions in the provision of 340B-discounted products to contract pharmacies previously described. Nevertheless, this study is a major contribution to the literature on the 340B program because it is the first, to our knowledge, to quantify trends in claims prescribed by 340B-affiliated clinicians and filled in 340B pharmacies. The consideration of capture by 340B pharmacies, which differentiates this study from prior work, is critical because only a share of claims prescribed by 340B-affilaited clinicians is filled in 340B pharmacies, and, as we demonstrate, this share has changed dramatically over time.

## Conclusions

As shown in this cohort study, from 2013 to 2020 the share of Medicare Part D prescriptions prescribed by 340B-affiliated clinicians increased; however, the rate at which 340B-eligible prescriptions were filled at 340B pharmacies increased at a faster rate, driving the overall increase in claims subject to 340B discounts. Despite these trends, only half of 340B-eligible prescriptions are actually subject to the 340B discount. As policymakers consider changes to the 340B program, they should consider the distribution of 340B discounts across therapeutic classes and the effect of limiting 340B discounts on drug price growth.

## Notes

Supplement 1. **eTable 1.** Medicare Part D Percent 340B Claim Eligibility by Prescriber, Pharmacy, and Overall, by Therapeutic Class, 2013 and 2020 **eTable 2.** Medicare Part D Percent 340B Claim Eligibility by Prescriber, Pharmacy, and Overall, for High-Spend Drugs, 2013 and 2020

Supplement 2.
Data Sharing Statement
