# Supplementary material for: Trends in Proportion of Medicare Part D Claims Subject to 340B Discounts, 2013-2020
Source: JAMA Health Forum. 2023 Nov 17;4(11):e234091. doi: 10.1001/jamahealthforum.2023.4091 (PMC10656642; doi:10.1001/jamahealthforum.2023.4091)
Supplement: Supplement 1. — eTable 1. Medicare Part D Percent 340B Claim Eligibility by Prescriber, Pharmacy, and Overall, by Therapeutic Class, 2013 and 2020 eTable 2. Medicare Part D Percent 340B Claim Eligibility by Prescriber, Pharmacy, and Overall, for High-Spend Drugs, 2013 and 2020 [file jamahealthforum-e234091-s001.pdf]

Supplemental Online Content

Dickson S, Gabriel N, Hernandez I. Trends in proportion of Medicare Part D claims subject to 340B discounts, 2013-2020. *JAMA Health Forum*. 2023;4(11):e234091. doi:10.1001/jamahealthforum.2023.4091

eTable 1. Medicare Part D Percent 340B Claim Eligibility by Prescriber, Pharmacy, and Overall, by Therapeutic Class, 2013 and 2020

eTable 2. Medicare Part D Percent 340B Claim Eligibility by Prescriber, Pharmacy, and Overall, for High-Spend Drugs, 2013 and 2020

This supplemental material has been provided by the authors to give readers additional information about their work.

© 2023 Dickson S et al. *JAMA Health Forum.*

eTable 1. Medicare Part D Percent 340B Claim Eligibility by Prescriber, Pharmacy, and Overall, by Therapeutic Class, 2013 and 2020

Therapeutic Class	Claims Prescribed by 340B-Affiliated Clinician		340B-Eligible Claims Filled at a 340B Pharmacy		Overall 340B Claims	
	2013	2020	2013	2020	2013	2020
**Overall sample**	9.4%	19.3%	18.4%	49.9%	1.7%	9.6%
**Classes with >100k claims in 2020**						
Antivirals	21.0%	28.1%	27.1%	57.3%	5.7%	16.1%
Antineoplastic Targeted Therapy	13.2%	31.3%	17.0%	50.0%	2.2%	15.7%
Antinauseants	11.3%	22.9%	19.5%	52.2%	2.2%	12.0%
Antineoplastic Chemotherapy	9.4%	21.6%	18.3%	52.4%	1.7%	11.3%
Respiratory Therapy	10.5%	21.3%	19.2%	52.2%	2.0%	11.1%
Cardiac Agents	9.6%	22.7%	16.9%	48.6%	1.6%	11.0%
Antimalarials	10.2%	20.5%	18.6%	51.4%	1.9%	10.5%
Diuretics And Aquaretics	10.4%	21.1%	17.8%	49.4%	1.9%	10.4%
Hemostatic Modifiers	9.2%	21.8%	17.8%	47.5%	1.6%	10.3%
Hormones	9.6%	19.6%	19.5%	52.6%	1.9%	10.3%
Diabetes Therapy	10.2%	19.9%	18.9%	51.0%	1.9%	10.2%
Musculoskeletal	9.1%	19.4%	18.8%	52.0%	1.7%	10.1%
Analgesics	9.9%	18.7%	20.5%	53.8%	2.0%	10.1%
Vascular Agents	9.6%	19.8%	18.1%	50.3%	1.7%	10.0%
Antiarthritics	9.3%	18.7%	19.4%	51.9%	1.8%	9.7%
Nutrients & Supplements	9.3%	20.5%	17.4%	46.8%	1.6%	9.6%
Antihyperlipidemic Agents	9.2%	19.0%	18.1%	50.2%	1.7%	9.5%
Gastrointestinal	9.6%	19.2%	18.0%	49.3%	1.7%	9.5%
Anti-Infectives, Systemic	9.1%	17.8%	20.3%	53.1%	1.9%	9.4%
Genitourinary	9.0%	19.2%	17.6%	48.3%	1.6%	9.3%
Laxatives	9.8%	19.5%	16.7%	47.6%	1.6%	9.3%
Anti-Fungal Agents	8.7%	17.7%	19.4%	50.8%	1.7%	9.0%
Neurological/Neuromuscular Disorders	9.1%	19.3%	17.1%	46.2%	1.6%	8.9%
Thyroid Therapy	8.5%	18.0%	17.3%	48.9%	1.5%	8.8%
Psychotherapeutic Drugs	8.9%	18.1%	17.9%	48.0%	1.6%	8.7%
Sedatives & Hypnotics	7.7%	15.0%	20.4%	54.3%	1.6%	8.2%
Allergy/Cold Preparations	7.4%	16.2%	17.3%	48.8%	1.3%	7.9%
Dermatologicals	7.7%	15.6%	17.9%	47.5%	1.4%	7.4%
Ophthalmic Preparations	4.9%	11.6%	15.5%	46.2%	0.8%	5.4%
Antiseptics	8.0%	15.0%	15.5%	34.2%	1.2%	5.1%
**Classes with < 100,000 claims in 2020**						
Immunologic Agents	14.0%	30.2%	19.6%	51.3%	2.7%	15.5%
Smoking Deterrents	11.3%	24.8%	24.5%	58.2%	2.8%	14.4%
Parasympathetic Drugs	9.5%	25.7%	18.5%	53.3%	1.8%	13.7%
Amebacide/Trichomacides/Antibacterials	11.4%	22.2%	19.3%	55.0%	2.2%	12.2%
Antidiarrheals	10.8%	22.2%	19.7%	51.7%	2.1%	11.5%
Anesthetics	9.9%	23.2%	19.6%	47.2%	1.9%	10.9%
Otic Preparations	7.7%	17.3%	21.5%	52.7%	1.7%	9.1%
Vitamins	10.1%	17.6%	15.8%	47.6%	1.6%	8.4%
Hemorrhoidal Preparations	8.5%	16.6%	19.1%	48.5%	1.6%	8.0%
Anthelmintics	6.6%	15.2%	18.0%	44.8%	1.2%	6.8%
Hospital Solutions	11.9%	21.4%	17.9%	22.2%	2.1%	4.8%

© 2023 Dickson S et al. *JAMA Health Forum.*

eTable 2. Medicare Part D Percent 340B Claim Eligibility by Prescriber, Pharmacy, and Overall, for High-Spend Drugs, 2013 and 2020

Drug	Claims Prescribed by 340B-Affiliated Clinician		340B-Eligible Claims Filled at a 340B Pharmacy		Overall 340B Claims	
	2013	2020	2013	2020	2013	2020
Biktarvy		51.38%		61.09%		31.38%
Imbruvica		37.27%		73.95%		27.56%
Xtandi	15.57%	33.60%	27.39%	62.19%	4.27%	20.89%
Symbicort	9.61%	22.28%	19.92%	53.70%	1.91%	11.96%
Novolog	11.39%	23.67%	18.26%	48.18%	2.08%	11.40%
Lantus	11.71%	21.81%	20.00%	51.98%	2.34%	11.34%
Eliquis	11.24%	22.98%	19.19%	47.47%	2.16%	10.91%
Xarelto	8.85%	21.88%	19.52%	47.97%	1.73%	10.50%
Januvia	9.52%	19.03%	19.32%	47.88%	1.84%	9.11%
Myrbetriq	8.82%	19.41%	19.32%	46.40%	1.70%	9.01%

© 2023 Dickson S et al. *JAMA Health Forum.*
