# Supplementary material for: Trends in Proportion of Medicare Part D Claims Subject to 340B Discounts, 2013-2020
Source: JAMA Health Forum. 2023 Nov 17;4(11):e234091. doi: 10.1001/jamahealthforum.2023.4091 (PMC10656642; doi:10.1001/jamahealthforum.2023.4091)
Supplement: Supplement 2. — Data Sharing Statement [file jamahealthforum-e234091-s002.pdf]

Data Sharing Statement

Dickson. Trends in Proportion of Medicare Part D Claims Subject to 340B Discounts, 2013-2020. *JAMA Health Forum*. Published November 17, 2023.
doi:10.1001/jamahealthforum.2023.4091

Data
Data available: No

Additional Information
Explanation for why data not available: Data were obtained under a data user agreement that does not allow data sharing
